# An unexpected case of *Borrelia garinii* liver infection

**DOI:** 10.1186/s12941-022-00506-6

**Published:** 2022-03-27

**Authors:** Pierre Duffau, Skander Korbi, Vivien Guillotin, Emilie Talagrand-Reboul, Armelle Ménard, Olivia Peuchant

**Affiliations:** 1 Centre Hospitalier Universitaire de Bordeaux, Service de médecine interne et immunologie clinique, 33000 Bordeaux, France; 2 Univ. Bordeaux, CNRS UMR 5164, Immuno ConcEpT, 33000 Bordeaux, France; 3 Centre Hospitalier Universitaire de Bordeaux, Service d’anatomo-pathologie, 33000 Bordeaux, France; 4 Université de Strasbourg, UR7290, Fédération de Médecine Translationnelle de Strasbourg, 67000 Strasbourg, France; 5 Hôpitaux Universitaires de Strasbourg, Institut de Bactériologie, Centre National de Référence des Borrelia, 67000 Strasbourg, France; 6 Centre Hospitalier Universitaire de Bordeaux, Laboratoire de Bactériologie, 33000 Bordeaux, France; 7 Univ. Bordeaux, INSERM, UMR1053 Bordeaux Research in Translational Oncology, BaRITOn, 33000 Bordeaux, France; 8 Univ. Bordeaux, INRAE, USC EA 3671, Infections humaines à mycoplasmes et chlamydiae, 33000 Bordeaux, France

**Keywords:** *Borrelia garinii*, 16S rDNA, Liver involvement, Kupffer cell hyperplasia, Warthin Starry stain

## Abstract

**Background:**

Lyme borreliosis is the most prevalent arthropod-borne infection in the Northern Hemisphere. In Europe, *Borrelia afzelii* is predominantly involved in cutaneous manifestations, *Borrelia* *garinii* and *Borrelia bavariensis* in neurological manifestations, and *Borrelia* *burgdorferi* sensu stricto in articular ones. Liver impairement is not classical in Lyme borreliosis. Diagnosis is currently mainly based on serological testing, and is challenging in immunocompromised patients.

**Case presentation:**

We report the first case of *B. garinii* infection revealed by liver involvement in an immunocompromised man. A 73-year-old man with marginal zone lymphoma, treated with bendamustine and rituximab, developed intermittent fever and inflammatory syndrome. Microbial investigations were all negative and FDG-PET showed complete remission of the lymphoma. Three months later, liver biopsy was performed and histology revealed spirochetes-like bacteria. Microbial diagnosis was performed by 16S rDNA sequencing, flagellin (*fla*B) gene sequencing and multi-locus sequence typing and identified *B. garinii*. The patient recovered successfully after a three weeks course of antibiotics. Diagnosis was challenging because *Borrelia* hepatic involvement is unusual and no *erythema migrans* nor tick bite were notified.

**Conclusion:**

This case highlights that unexplained fever and inflammatory syndrome in immunocompromised patients warrants specific investigations to identify bacteria such as spirochetes.

## Background

Lyme borreliosis is caused by the tick-borne spirochetes of the *Borrelia burgdorferi* sensu lato (s.l.) complex and is the most prevalent arthropod-borne infection in the Northern Hemisphere. Its incidence increased over the last few decades in many European countries, including France [1, 2]. This multisystem disease is caused by infection with spirochaetal bacteria of the *B. burgdorferi* s.l. complex. These bacteria are transmitted to humans and other vertebrate hosts via the bite of infected *Ixodes* spp. ticks, mainly *Ixodes ricinus* in Europe [3].

Five Borrelia species are mainly pathogenic to humans: *Borrelia afzelii*, *Borrelia burgdorferi* sensu stricto (s.s.), *Borrelia garinii*, *Borrelia bavariensis* and, less often reported, *Borrelia spielmanii* [3]. All five species are present in Europe, although *B. afzelii* and, to a lesser extent, *B. garinii* predominate, whereas *B. burgdorferi* s.s. is the main species responsible for Lyme borreliosis in North America [3].

*Borrelia* infection in humans can cause a range of clinical features. Patients may present with a variety of symptoms, which can vary according to the stage of the disease and the level of bacterial dissemination through the blood and tissues [3]. Localized infection is typically manifested by *erythema migrans* skin lesion. It appears in the early stages of the disease and presents at the initial site of inoculation. Early disseminated disease is characterized by neuroborreliosis (mainly presents as meningoradiculitis and cranial nerve palsy), Lyme arthritis, or more rarely, multiple *erythema migrans*, borrelial lymphocytoma or Lyme carditis. Late Lyme borreliosis usually manifests as chronic Lyme arthritis, acrodermatitis chronica atrophicans, and late neurological manifestations [4]. Of the various objective clinical presentations, *erythema migrans* is the most common.

Infection with certain *Borrelia* spp. has been associated with specific disseminated clinical manifestations. Current knowledge is that *B. afzelii* is predominantly involved in cutaneous manifestations in Europe, is often found in the localized incipient form of the infection and in acrodermatitis chronica atrophicans, *B. garinii* and *B. bavariensis* in neurological manifestations, and *B. burgdorferi* s.s. in articular ones [3].

Liver impairement is not classical in Lyme borreliosis. We here report the first case of liver involvement associated with *B. garinii* infection.

## Case presentation

**Fig. 1:**
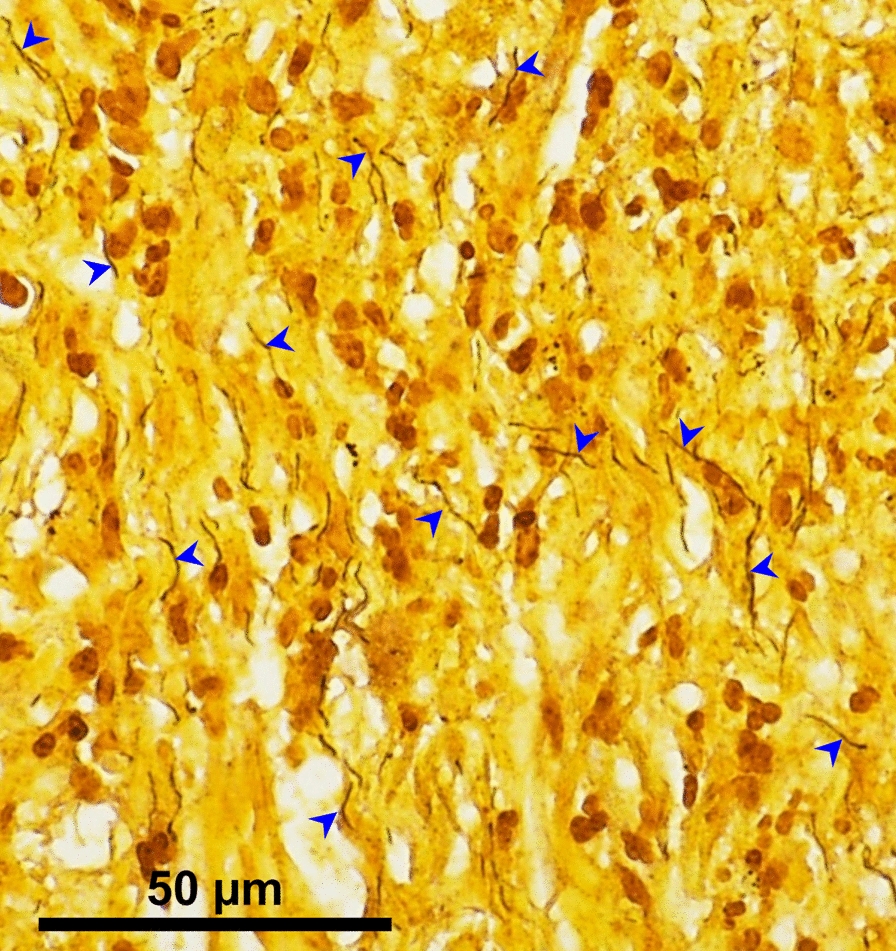
Images of human liver biopsy specimens. Warthin Starry silver nitrate staining showing spirochete-like bacteria (blue arrowheads) (original magnification × 400)

In January 2020, a 73-year-old French man with no medical history, was diagnosed with a marginal zone lymphoma. He received chemotherapy consisting of bendamustine and rituximab. After the fourth cycle of chemotherapy, he developed inflammation with elevated serum C-reactive protein (150 mg/L) without any clinical symptoms. No abnormal findings were observed on physical examination. Fluorodeoxyglucose (FDG) positron emission tomography (PET) demonstrated lymphoma complete remission. He nevertheless received two additional cycles of chemotherapy until May 2020. Then, he developed intermittent fever and nocturnal sweat. He was admitted to hospital in July 2020. Except a temperature of 38.7 °C, physical examination was normal. No tick bite or *erythema migrans* was notified. Laboratory tests revealed the following: alkaline phosphatase 360 U/L, ALAT 64 U/L, ASAT 67 U/L, white blood cell count 8.4 g/L. His immunity state was obviously compromised with deep lymphopenia (0.54 g/L) and hypogammaglobulinemia (2.6 g/L). Computerized tomography-scan as well as cardiac echography were normal. Serological tests for *B. burgdorferi* s.l., *Francisella* spp., *Bartonella* spp., *Brucella* spp., *Rickettsia* spp. and *Coxiella* spp. were negative. Blood and urine culture, PCR targeting *Tropheryma whipplei* in blood as well as tests for anti-nuclear antibodies and rheumatoid factor, were all negative. Bone marrow biopsy and a new FDG-PET confirmed that lymphoma was still in complete remission. As patient’s state was worsening, we started prednisone at the dose of 0.5 mg/kg with dramatic but only transient improvement. During the three months follow-up, the patient presented slightly elevated levels of cholestasis parameters that were initially attributed to chronic inflammation. As fever persisted, liver biopsy was performed. Histology showed significant sinusoidal dilation (peliosis) and a rich inflammatory infiltrate composed predominantly of neutrophils. The latter were intra-sinusoidal and infiltrated the portal spaces and bile ducts (neutrophilic cholangitis/cholangiolitis). Additional findings included Kupffer cell hyperplasia with hemophagocytosis and extramedullary hematopoiesis. Warthin Starry stain highlighted spirochetes-like bacteria within hepatic sinusoids (Fig. 1).

**Fig. 2:**
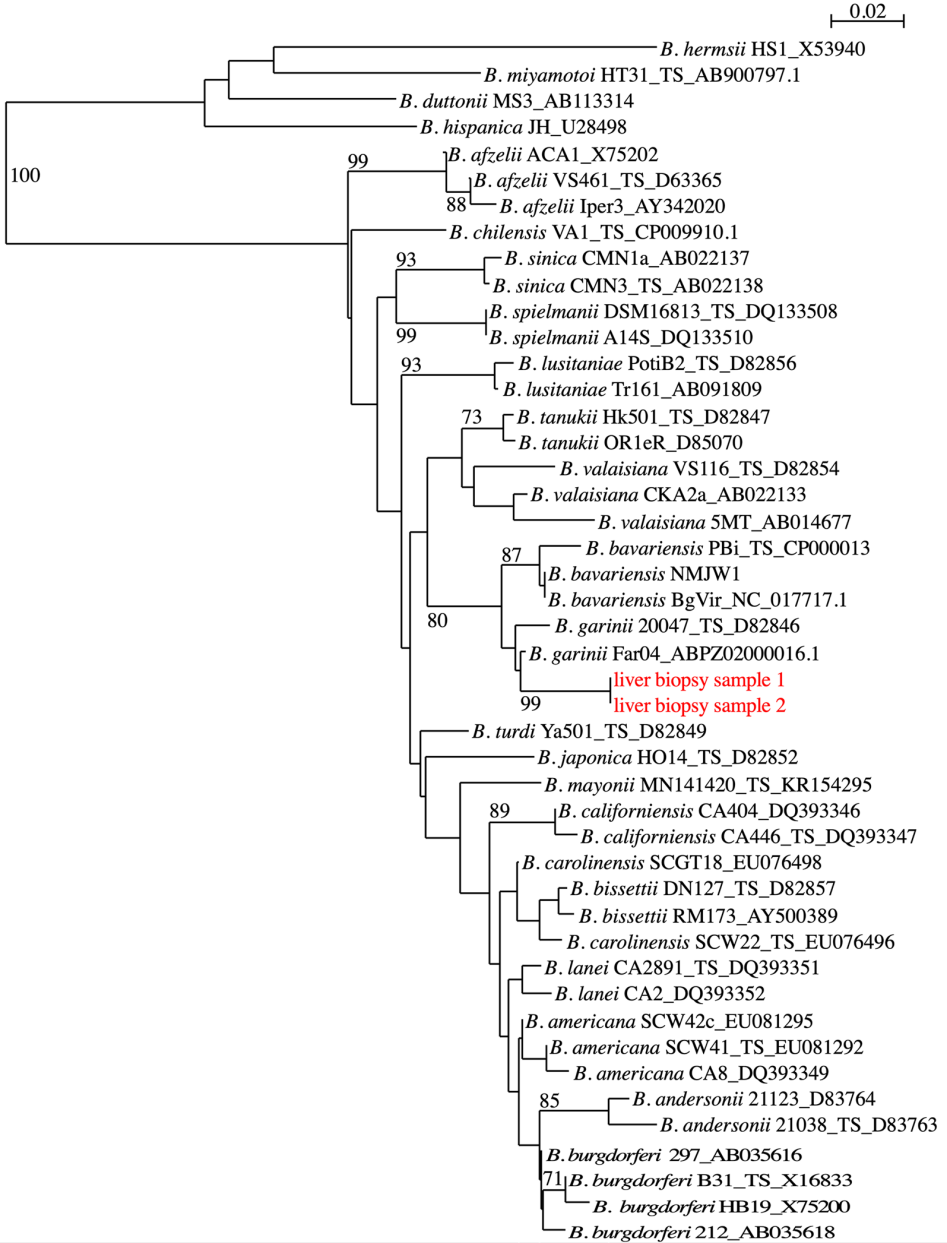
Phylogenetic tree of Borrelia taxa based on flagellin gene (158 nt). DNA extracts from two different areas of the liver biopsy were analyzed (indicated in red). The phylogenetic analyses were generated with the BioNeighbor-Joining method. The phylogeny presented is based on the alignment of 158-bp sequences of *fla*B gene from 44 reference sequences representing the current known diversity in the genus Borrelia and the sequence amplified in the case report presented herein. The percentage of replicate trees in which the associated taxa clustered together in the bootstrap test (1,000 replicates) is shown next to the branches. The tree is not rooted and drawn to scale, with branch lengths in the same units as those of the evolutionary distances used to infer the phylogenetic tree. The evolutionary distances were computed using the Kimura 2-parameter method and are represented in the units of the number of base substitutions per site. Evolutionary analyses were conducted using SeaView. All sequences are labeled by species, strain name, and GenBank accession number. *TS* type strain

Standard bacterial cultures of liver biopsy were negative. A broad-range 16S rRNA gene-based PCR performed on a DNA extract from liver biopsy led to a 1457 bp sequence (GenBank accession number: MZ227021) that showed 100% similarities with the sequence of *B. garinii* strain 20047 (GenBank accession number CP028861.1). To confirm the species *B. garinii*, DNA extract of liver biopsy was sent to the French National Reference Center (NRC) for Borrelia (Strasbourg, France). Liver biopsy was also sent to the NRC for borrelial culture and PCR. Specific PCR for *B. burgdoferi* s.l. targeting the *fla*B gene turned out strongly positive (Cycle threshold value 30). Identification at the species level was confirmed by the phylogenetic analysis of the *fla*B gene, which showed that the isolate was clustered within *B. garinii* species (Fig. 2).

Multilocus sequence typing (MLST) was performed on eight housekeeping genes [5]. Complete allelic profile was obtained for five genes: *clp*A#43, *nif*S#30, *pep*X#90, *pyr*G#87, *rec*G#36 (www.pubmlst.org/borrelia). We obtained a new allele for the *clp*X gene, closest to *clp*X#28 (one nucleotide difference). All of these sequences were confirmed to originate from the species *B. garinii* by comparison to sequences in the MLST database.

After establishing this diagnosis, we confirmed that patient was truly seronegative two months after the onset of symptoms, with two different first-step serological tests (Liaison® Borrelia IgG and IgM, Diasorin and Enzygnost IgG and IgM ELISA, Siemens).

The patient was treated with ceftriaxone (2 g/day) for three weeks. The patient recovered successfully, and no signs of recurrence was observed in the following six months.

## Discussion

In humans, only five cases of clinical liver damage have been reported to date in Lyme borreliosis [6–10]. Chavanet et al. reported a case of febrile granuloma hepatitis in a 46-year-old man with no medical history [6]. Goellner et al. described a case of hepatitis in a 73-year-old woman that appeared to be the result of direct tissues invasion by the spirochete [8]. Histopathology of liver biopsy showed ballooning of hepatocytes, marked hepatocyte mitotic activity, prominent microvesicular fat, Kupffer cell hyperplasia, and sinusoidal infiltration by mononuclear cells and polynuclear neutrophils. Dieterle silver-staining showed spirochetes within hepatic sinusoids and parenchyma, but molecular identification was not performed and culture remained negative. Dadamessi et al. described a case of liver injury in a 71-year-old man who presented with febrile jaundice; liver biopsy revealing a non-specific sinusoidal inflammatory infiltrate without necrosis [7]. Zanchi et al. reported a case of Lyme borreliosis with necrotizing granulomas and eosinophilic infiltration of the hepatic parenchyma [9]. Then, Middelveen et al. described a case of granulomatous hepatitis associated with chronic Lyme borreliosis in a 53-year-old woman [10]. Spirochetes were observed within parenchyma of the liver biopsy tissue on histological examination, and identification of *B. burgdoferi* s.s was performed from positive blood cultures using molecular methods [10]. Although it is possible to see spirochetes on liver biopsy in patients with Lyme borreliosis and hepatitis, in the majority of these cases, no organisms are identified and diagnosis relies on positive serological tests. Spirochetes cannot be detected by Gram staining, whereas Warthin Starry staining revealed them in liver biopsy in the present case, allowing the subsequent diagnosis of *B. garinii* infection by 16S rRNA gene-based molecular methods. This is the third case where the spirochete was directly demonstrated in liver biopsy.

The pathogenesis of liver injury in patients with Lyme borreliosis includes an interplay of direct hepatic invasion by the spirochete and immunologic responses. Lyme borreliosis can result in a variety of histologic abnormalities in the liver, in particular, sinusoidal infiltration by a mixed inflammatory infiltrate [8]. Rarely, *B. burgdoferi* s.l. can result in granulomatous hepatitis.

*B. burgdoferi* s.l. strains are not known to produce toxins. Most tissue damage seems to result from host inflammatory reactions. The intensity of inflammatory response varies according to the *Borrelia* species that causes an infection [11].

In the present case, the patient did not remember a tick bite or *erythema migrans*, as also described in other cases [6, 7, 9]. *Erythema migrans* is the most common skin finding seen in more than 80% of patients with early Lyme borreliosis [12]. It typically develops 7 to 14 days (range, 3 to 30) after tick detachment [13]. The *erythema migrans* lesions are often found in or near the armpit, inguinal region, or popliteal fossa but can occur in any part of the body.

Gastrointestinal signs and symptoms are common in the early stages of the disease. In a study of 314 patients, approximately 10% of the patients had symptoms that were suggestive of hepatitis [14]. In another study, 27% of the patients had subclinical hepatitis during the early stages of disease [15]. Patients with early disseminated Lyme borreliosis are more likely to have abnormal liver function test finding than are patients with localized disease [16]. However, elevations in aspartate aminotransferase may indicate Lyme disease-associated myositis in some patients and may not be related to underlying hepatic injury.

In our patient, serological assays that detect antibodies for *B. burgdorferi* s.l. were negative while it is generally accepted that patients with disseminated Lyme borreliosis with symptoms for more than six weeks usually reveal positive *Borrelia* serology [17]. This was explained by the lymphopenia and the reduction of total IgA/IgM/IgG concentrations, in agreement with other observations [18]. Cultivation and isolation from clinical material is a golden standard for confirmation of *Borrelia* infection [19]. However, many factors influence in vitro* Borrelia* growth such as medium ingredients and its pH, temperature of incubation, the presence of contaminants, sample’s cell density, number of *Borrelia* strains in the sample, antecedent antibiotic therapy or capacity of particular *Borrelia* species to grow [17, 19, 20]. Indeed, *Borrelia* culture is impractical for routine clinical use [17, 19, 20]. In the present case, diagnosis was established using molecular methods, directly performed on liver biopsy, and permitted the official identification of the bacterium. The gold standard for genotyping of *B. burgdorferi* s.l. nowadays is MLST, which undergo slow evolution and show nearly neutral variation [5]. Of interest, MLST analysis of *B. garinii* isolates from bird-derived ticks, questing ticks and humans revealed that there was little overlap among genotypes from different continents, no geographical structuring within Europe, and no evident association pattern detectable among *B. garinii* genotypes from ticks feeding on birds, questing ticks or human isolates [21]. This provides supporting evidence that birds act as important reservoirs for *B. garinii* and are a main source of infection of this species to ticks and ultimately humans (through the bite of an infected tick).

## Conclusion

This case illustrates the potential for seronegative Lyme borreliosis in patients with immunomodulatory therapy, and the need for further investigations to avoid missing or delaying the accurate diagnosis. Diagnosis can be difficult because *erythema migrans* is not always present, and *Borrelia* liver involvement rarely occurs.

## Data Availability

Not applicable.

## Abbreviations

FDG-PET: Fluorodeoxyglucose positron emission tomography
MLST: Multilocus sequence typing
